# Biases in read coverage demonstrated by interlaboratory and interplatform comparison of 117 mRNA and genome sequencing experiments

**DOI:** 10.1186/1471-2105-13-S6-S4

**Published:** 2012-04-19

**Authors:** Ekaterina E Khrameeva, Mikhail S Gelfand

**Affiliations:** 1 Institute for Information Transmission Problems, Russian Academy of Sciences, 19 Bolshoy Karetny per., Moscow, 127994, Russia; 2 Faculty of Bioengineering and Bioinformatics, Moscow State University, 1-73 Leninskie gory, Moscow, 119991, Russia

## Abstract

High-throughput sequencing of whole genomes and transcriptomes allows one to generate large amounts of sequence data very rapidly and at a low cost. The goal of most mRNA sequencing studies is to perform the comparison of the expression level between different samples. However, given a broad variety of modern sequencing protocols, platforms and versions thereof, it is not clear to what extent the obtained results are consistent across platforms and laboratories. The comparison of 117 human mRNA and genome high-throughput sequencing experiments performed on the Illumina and SOLiD platforms at 26 institutions all over the world demonstrated high dependency of the gene coverage profiles on the producing laboratory. Gene coverage profiles showed laboratory-specific non-uniformity that survived the 3'-bias correction and mappability normalization, suggesting that there are other yet unknown mRNA-associated biases.

## Background

Next-generation sequencing technologies have completely transformed the field of genetics, making it possible to generate large amounts of sequence data very rapidly and at a low cost. High-throughput sequencing of whole genomes and transcriptomes has become a major focus of modern biology as DNA sequencing is now available to many more projects, and even single research groups. As the performance of platforms or versions may differ, it is not clear to what extent the obtained results are consistent across platforms or versions thereof, or even between different laboratories using the same equipment [1].

Many efforts have been made to understand and overcome the biases inherent in the next-generation sequencing technology. On the Illumina platform, regions of elevated GC content have higher read coverage; sequencing errors occur preferentially at the 3'-end of reads; sequences preceding error positions are G-rich; the transversions *G → T *and *A → C *are the most frequent substitutions; quality scores underestimate the true error rate for high quality values and overestimate the true error rate for low quality values [2]. It has been shown that the distributions of the sequenced nucleotides change across the positions of the reads and this bias influences the uniformity of the read location along expressed transcripts [3,4]. Also, there are PCR biases over-amplifying identical cDNA fragments [5]; mappability bias leading to lower coverage of regions with low sequence complexity; non-hydrolysis bias increasing levels of 5'-termini in the sequenced pool [6]. mRNA sequencing may be influenced by contamination by non-processed or under-spliced transcripts leading to visible intron coverage; mRNA degradation when RNAs are selected by polyA leading to the higher coverage of the 3'-end; influence of RNA secondary structure on fragmentation.

Here, we compared results of 117 human mRNA and genome high-throughput sequencing experiments performed on the Illumina and SOLiD platforms of all generations at 26 institutions all over the world to demonstrate the existence of systematic biases that can potentially affect gene coverage profiles. The gene coverage profiles are important for widely applied differential expression studies because gene coverage non-uniformity can lead to wrong estimations of expression levels of different exons, for example at the beginning or at the end of the gene. We observed high dependency of the gene coverage profiles on the producing laboratory, with most Illumina mRNA datasets showing existence of a systematic bias, while the genome datasets were not biased significantly.

## Results

Starting with the raw data, we calculated coverage profiles that are normally used to determine biologically relevant parameters such as gene expression or exon inclusion rates. Only single-exon genes filtered for high coverage (178 genes, see Methods for details) were considered to make gene coverage profiles comparable between mRNA sequencing experiments for different tissues that may produce alternative splice isoforms, and also to allow for the comparison of genomic and transcriptomic data.

**Figure 1:**
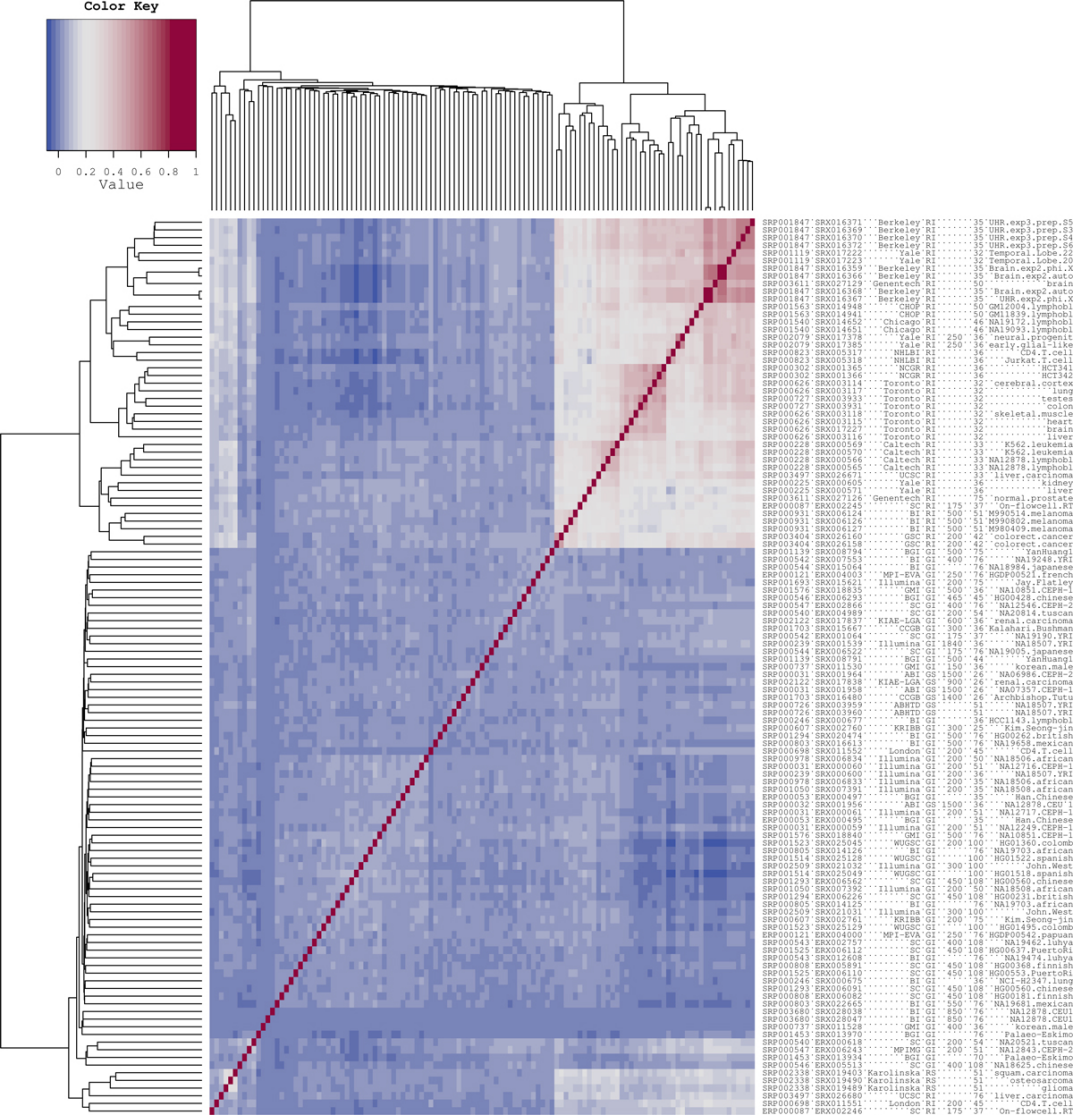
**Correlation of per-nucleotide coverage profiles between all pairs of sequencing experiments**. The heat map colors represent the Pearson correlation coefficients. Shades of blue correspond to the interval (-0.1, 0.2); shades of red correspond to (0.2, 1.0). Individual experiments are clustered by gene coverage. The labels contain the following information about experiments: SRA study ID; SRA experiment ID; institution short name; genome ("G") or transcriptome ("R") sequencing; platform ("I" stands for Illumina, "S" for SOLiD); fragment length if reads are paired; read length; individual ID, nationality, cell line and/or tissue. Additional information about experiments can be found in Additional file 6.

**Figure 2:**
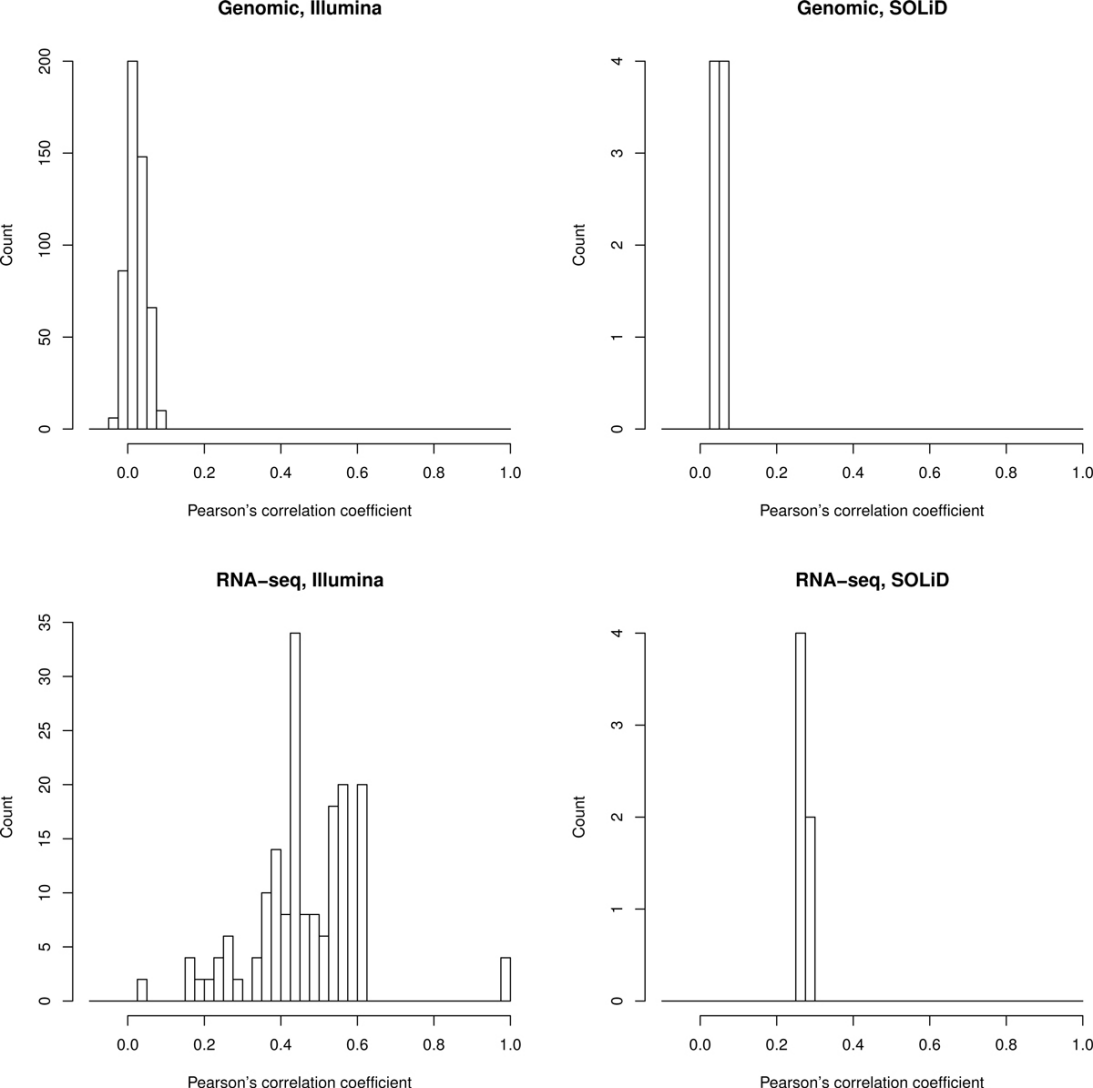
**Distribution of Pearson's correlation coefficients in the same laboratory**. Pearson's correlation coefficients were calculated between all possible pairs of different experiments of the same laboratory independently for each single-exon gene coverage profile and averaged by such genes.

**Figure 3:**
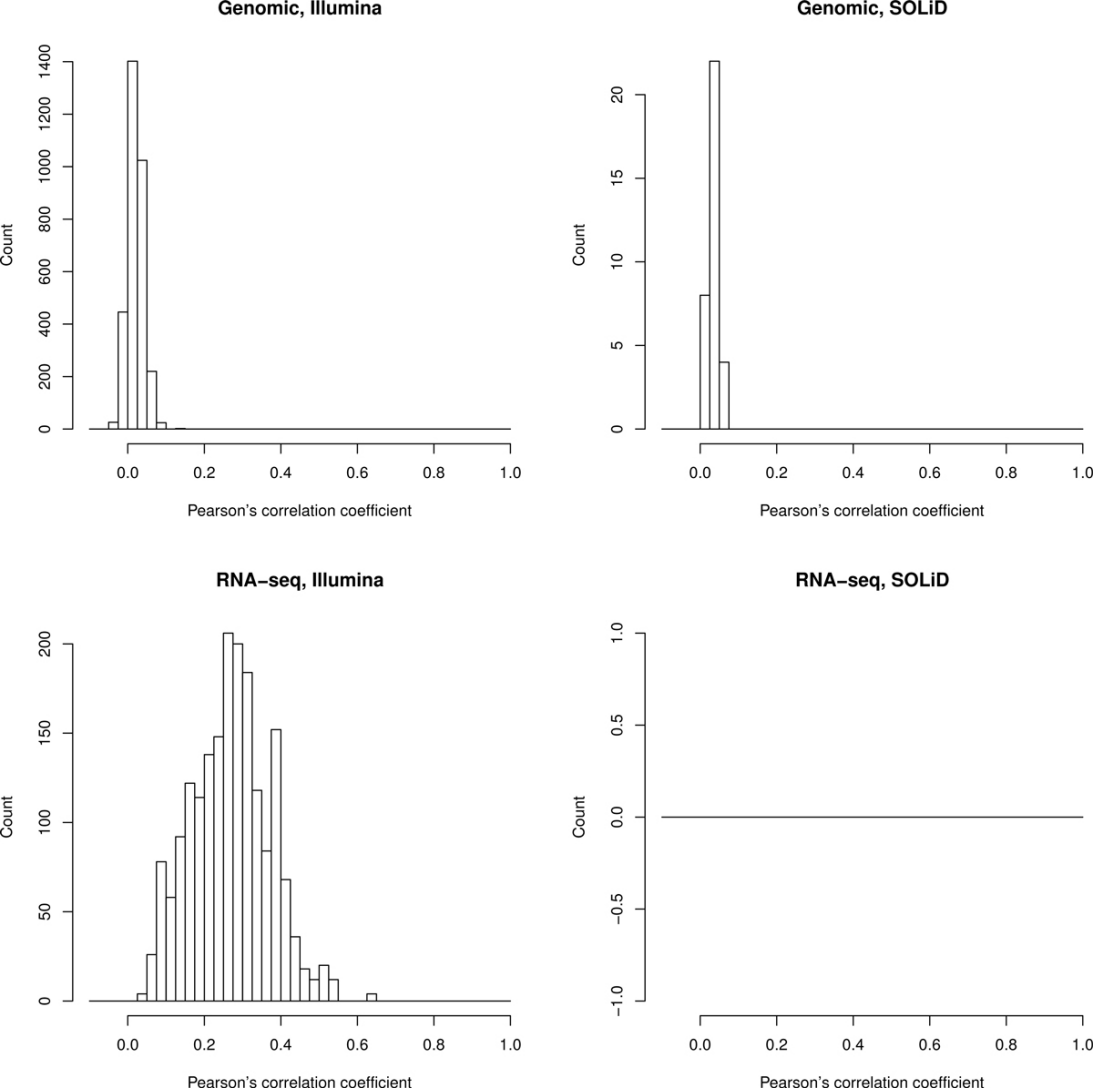
**Distribution of Pearson's correlation coefficients between laboratories**. Pearson's correlation coefficients were calculated between all possible pairs of diffierent experiments between laboratories independently for each single-exon gene coverage profile and averaged by such genes.

Clustering of experiments (Figure 1) showed that mRNA sequencing experiments coming from the same laboratory tend to have rather similar gene coverage profiles even if very different tissues such as brain and liver were sequenced (average correlation of profiles for the same gene in different experiments of the same laboratory 0.46 ± 0.14 for the Illumina RNA data, Figure 2). At the same time, sequencing of transcriptomes in different laboratories, even from the same tissue and on identical platforms, yielded quite different gene coverage profiles (average correlation of profiles for the same gene between laboratories 0.27 ± 0.10, Figure 3). We applied the Wilcoxon rank sum test to check if the average correlation of profiles is significantly higher in the same laboratory than between laboratories, and found p-value to be less than 2.2*e*^-16^, meaning that the gene coverage profiles are indeed more similar within the same laboratory than between different laboratories.

The genome sequencing experiments do not produce such a clear picture. While some of them cluster by laboratory, others do not. The SOLiD experiments all cluster together. The mRNA sequencing experiments performed on the SOLiD platform are distant, by gene coverage profiles, from other mRNA sequencing experiments, as well as from the genome sequencing experiments, including those performed on SOLiD.

The clustering procedure was repeated for the unfiltered set of single-exon genes (1074 genes, see Additional file 1), which demonstrated generally the same result as the filtered one. Hence our observations do not depend on the filtering criterion.

Most Illumina mRNA datasets show coverage bias, with 3' gene termini, on average, covered higher than 5'-termini, as demonstrated by linear fitting of averaged coverage plots for all genes in a single experiment (see Additional files 2 and 3). On the contrary, in all SOLiD mRNA datasets 5' gene termini are covered higher than 3'-termini. To eliminate the 5' - 3' coverage non-uniformity, we normalized all datasets according to their linear fitting models and re-clustered the experiments (see Additional file 4). No significant difference was observed between dendrograms corresponding to the initial and normalized datasets. Normalized mRNA experiments cluster by laboratory as strongly as the initial ones (average correlation of profiles from one laboratory 0.43 ± 0.21, and between laboratories 0.25 ± 0.14, respectively).

**Figure 4:**
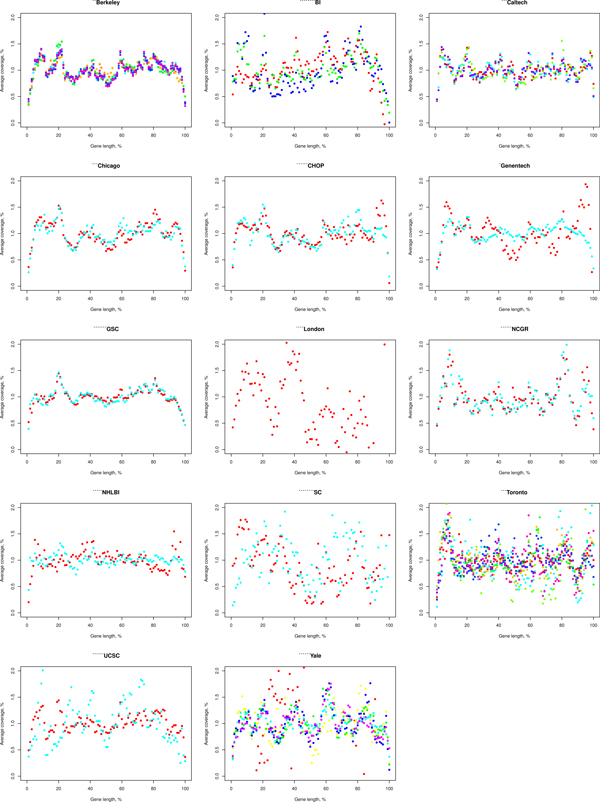
**Single-exon gene coverage distribution over gene length**. The distribution was calculated after normalization for 5' - 3' coverage bias. Points of different color represent different experiments grouped by laboratory.

There are some gene regions that are covered higher than the others, and their relative positions in genes are unique for each laboratory (Figure 4). This effect can not be explained by the read length, which is unique for a laboratory in most cases, as experiments do not tend to cluster by read length. We tried to overcome this bias by normalizing for read mappability, assuming that regions with lower sequence complexity tend to have lower sequence coverage. For each read length *L*, represented in the initial dataset, a mappability profile was produced by extraction of *L*-mer sequences beginning at each genomic position and their alignment to the reference genome with exactly the same parameters as the initial dataset. However, the normalization for the calculated mappability profiles also resulted in the same coverage non-uniformity effect (see Additional file 5).

## Discussion

The observed high dependency of the gene coverage profiles on the producing laboratory demonstrates that comparisons of sequencing results are difficult. This problem is crucial for mRNA sequencing experiments as the goal of most such studies is to compare the expression levels in various tissues, diseased and healthy individuals, case and control experiments, etc. As the genome sequencing experiments do not cluster distinctly by laboratory, they are most likely not biased significantly, whereas the transcriptome sequencing experiments demonstrate existence of a systematic bias that could be caused by the influence of the RNA secondary structure on the sample preparation and sequencing.

The 5' - 3' coverage non-uniformity has been observed before [7,8] and can be traced back to specifics of the random hexamers or oligo(dT) preparation protocols. Studies involving comparative analysis of sequencing data produced by the same laboratory would be largely unaffected by such artefacts (average correlation 0.46 ± 0.14), whereas biological implications of wider comparisons may require additional controls and normalization procedures (average correlation 0.27 ± 0.10).

Normalization for 5' - 3' coverage bias did not result in significant improvements, neither the mappability normalization. After normalization, some gene regions are still covered higher than the others, with their relative positions in genes unique for each laboratory, meaning that there may be other mRNA-associated laboratory-specific biases of an unknown origin.

## Materials and Methods

The data were retrieved from the NCBI Sequence Read Archive (SRA, http://www.ncbi.nlm.nih.gov/Traces/sra). In the SRA, the data are organized into sets of experiments that result from a single study, performed in one lab, presumably following the same protocol. Each experiment in a study contains one or more sequencing runs. Experiments that had the total amount of sequenced data exceeding 500 million bases (Mb) and were publicly available as of 10th of October, 2010 were selected for further analysis. For each experiment, several runs were retrieved to compile a dataset of about 1.5 billion sequenced bases (Gb). Studies SRP001106, SRP001699, SRP001734, SRP002009, and SRP002881 were excluded as the size of each of their runs exceeded 5 Gb.

Reads were aligned to the reference human genome (version hg19) with the program bowtie [9]. The number of allowed mismatches depended on the read length: one mismatch for reads shorter than 26 nucleotides (nt), two mismatches for the interval 26-50 nt, and three mismatches for reads longer than 50 nt. Reads produced by the Illumina platform were mapped in the base space and reads produced by SOLiD were mapped in the color space. Reads that mapped to multiple locations in the human genome were discarded.

Per-nucleotide gene coverage was calculated for each position of the gene as the total number of reads spanning over this position. For each gene, the Pearson correlation coefficient of the coverage profiles was calculated between all possible pairs of sequencing experiments. The coverage profiles were not preliminary normalized by the overall read count for each experiment because the Pearson correlation coefficient does not depend on the scaling factor.

Genes that were covered by reads in less than 90% of experiments were discarded. 178 of 1074 single-exon protein-coding genes survived this filtration. The correlation coefficient was averaged over these genes for each pair of sequencing experiments, which were clustered by the UPGMA method [10] in the R environment [11].

## Competing interests

The authors declare that they have no competing interests.

## Authors' contributions

MSG conceived the study. EEK developed the methods, performed the analysis, and drafted the manuscript. MSG reviewed and revised the manuscript.

## Supplementary Material

Additional file 1 **Supplementary figure S1**. Correlation of per-nucleotide coverage profiles between all pairs of sequencing experiments for an unfiltered set of single-exon genes (1074 genes). All notations are as in Figure 1. Click here for file

Additional file 2 **Supplementary figure S2**. Single-exon gene coverage distribution averaged over genomic/mRNA sequencing experiments on Illumina/SOLiD platforms. Click here for file

Additional file 3 **Supplementary figure S3**. Distribution of linear model fitting coefficients calculated for gene coverage profiles and averaged over genomic/mRNA sequencing experiments on Illumina/SOLiD platforms. Click here for file

Additional file 4 **Supplementary figure S4**. Correlation of per-nucleotide coverage profiles between all pairs of sequencing experiments normalized for the 5' - 3' coverage non-uniformity. Notations are similar to those in Figure 1. Click here for file

Additional file 5 **Supplementary figure S5**. Single-exon gene coverage distribution over gene length after normalization for mappability profiles. Points of different color represent different experiments grouped by laboratory. Click here for file

Additional file 6 **Supplementary figure S6**. Additional information about 117 compared mRNA and genome high-throughput sequencing experiments. Experiments are clustered by similarity of single-exon gene coverage profiles as in Figure 1. Click here for file
