# Supplementary figures and images for: Biases in read coverage demonstrated by interlaboratory and interplatform comparison of 117 mRNA and genome sequencing experiments
Source: BMC Bioinformatics. 2012 Apr 19;13(Suppl 6):S4. doi: 10.1186/1471-2105-13-S6-S4 (PMC3358657; doi:10.1186/1471-2105-13-S6-S4)

**Genomic, Illumina**

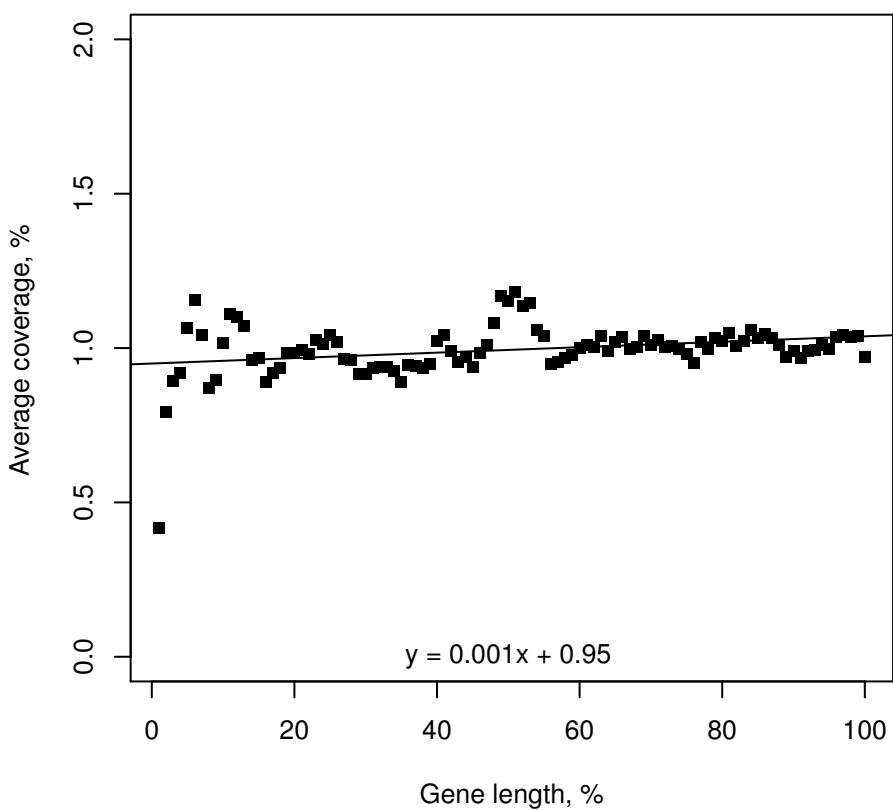

**Genomic, SOLiD**

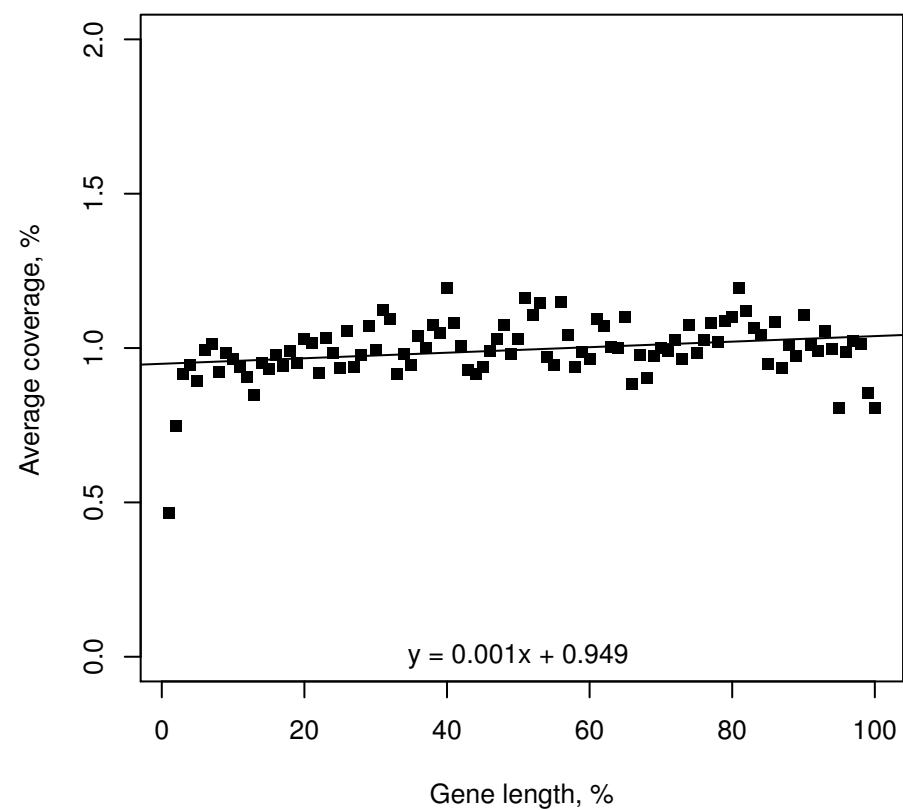

**RNA-seq, Illumina**

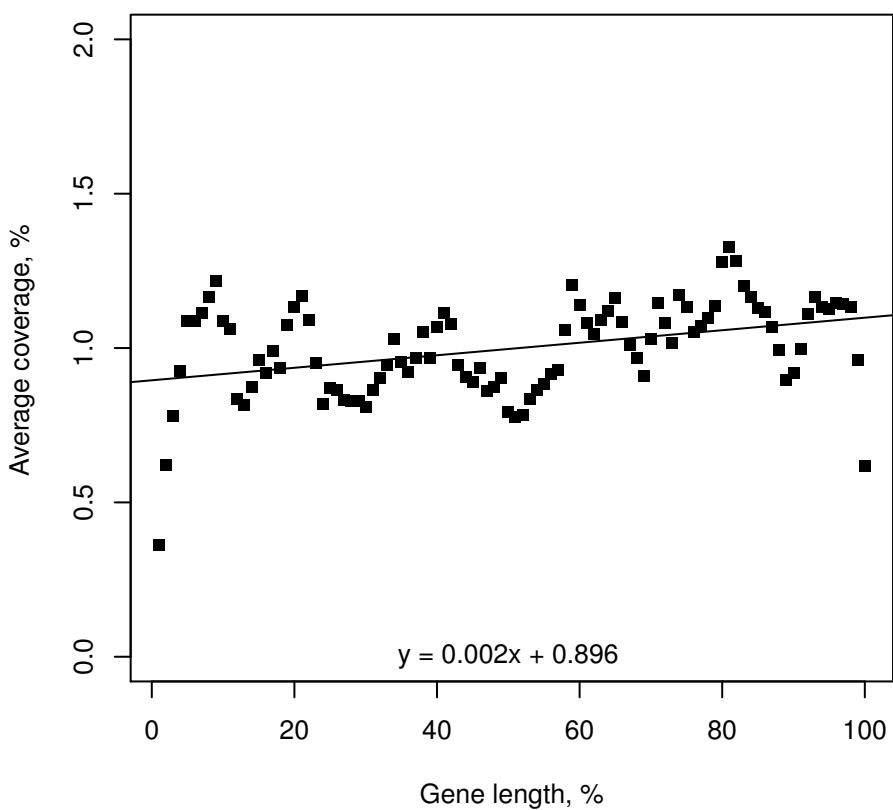

**RNA-seq, SOLiD**

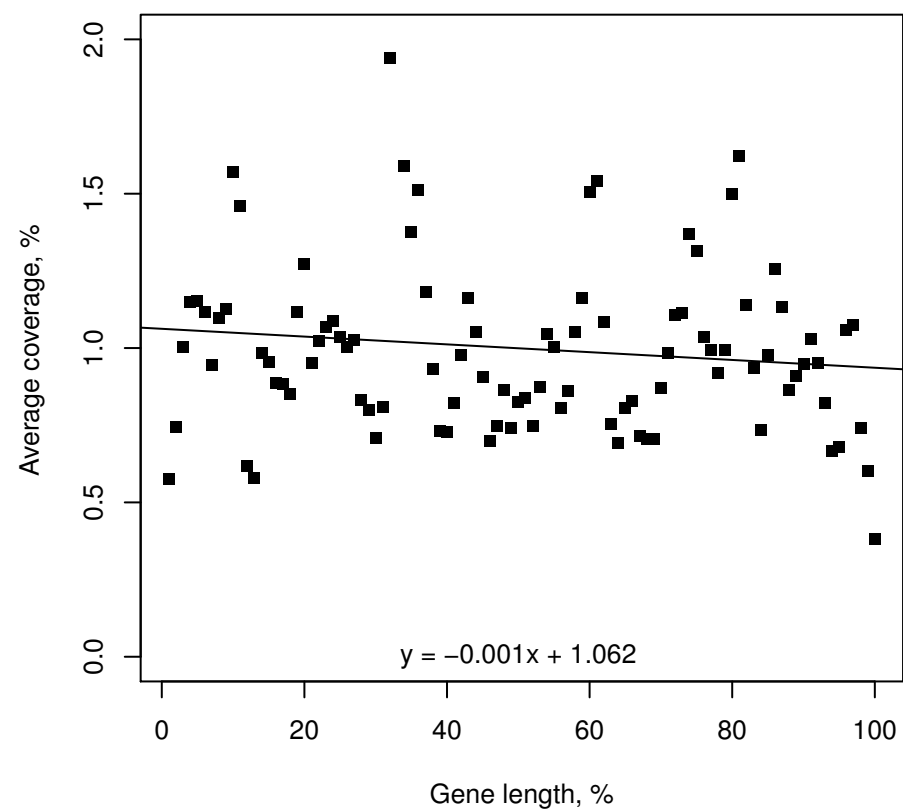

Supplement: Additional file 2 — Supplementary figure S2. Single-exon gene coverage distribution averaged over genomic/mRNA sequencing experiments on Illumina/SOLiD platforms. [file 1471-2105-13-S6-S4-S2.pdf]

**Genomic, Illumina**

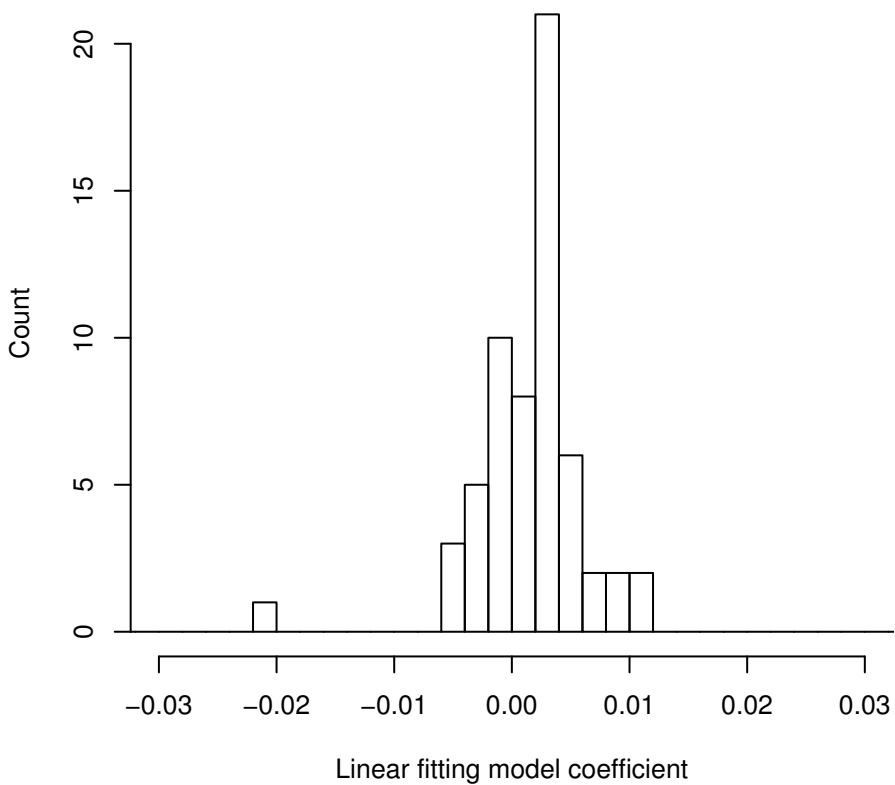

**Genomic, SOLiD**

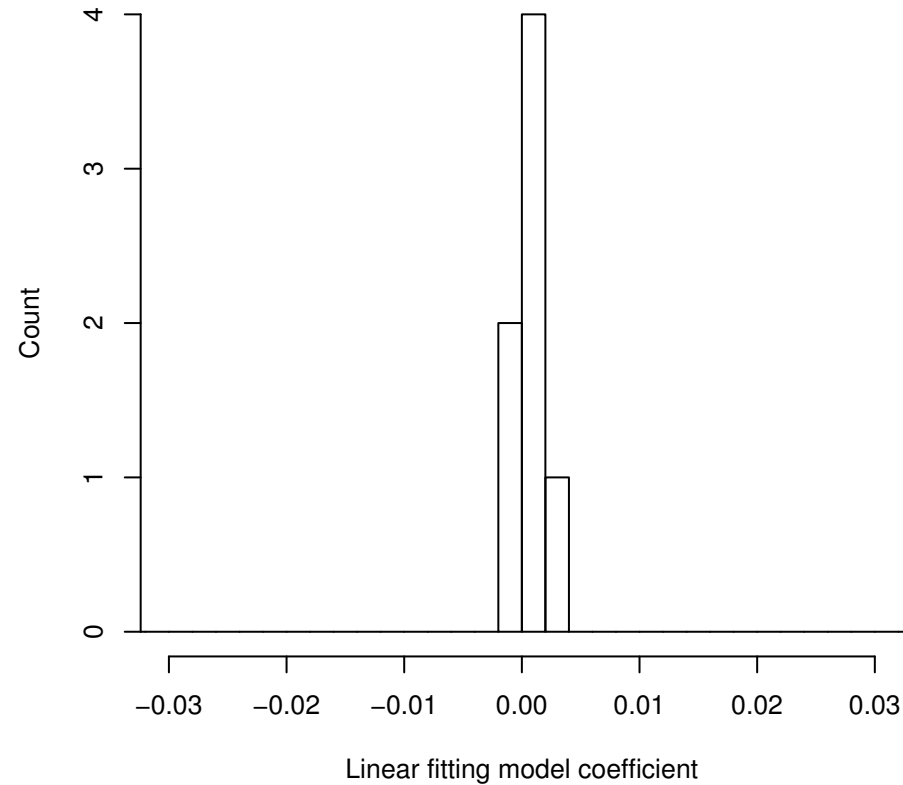

**RNA-seq, Illumina**

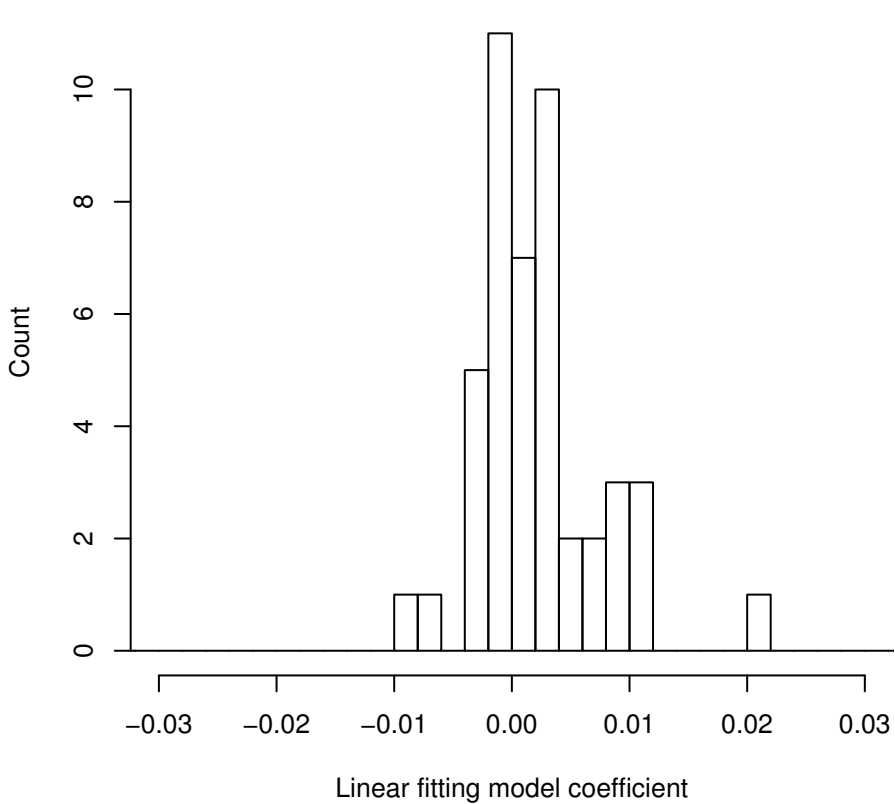

**RNA-seq, SOLiD**

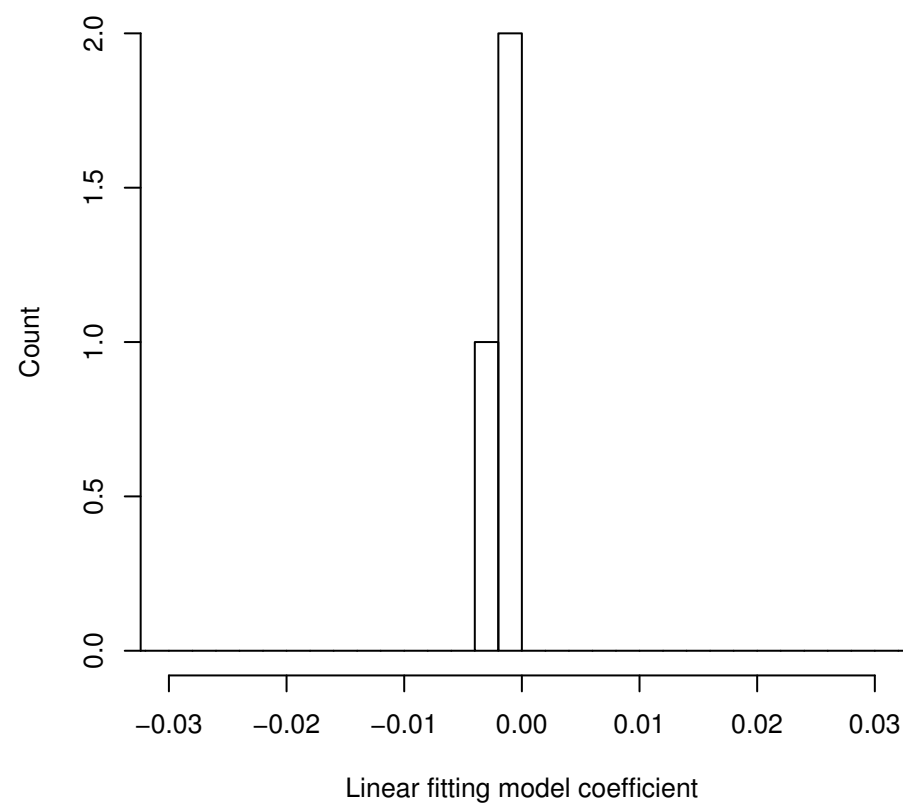

Supplement: Additional file 3 — Supplementary figure S3. Distribution of linear model fitting coefficients calculated for gene coverage profiles and averaged over genomic/mRNA sequencing experiments on Illumina/SOLiD platforms. [file 1471-2105-13-S6-S4-S3.pdf]

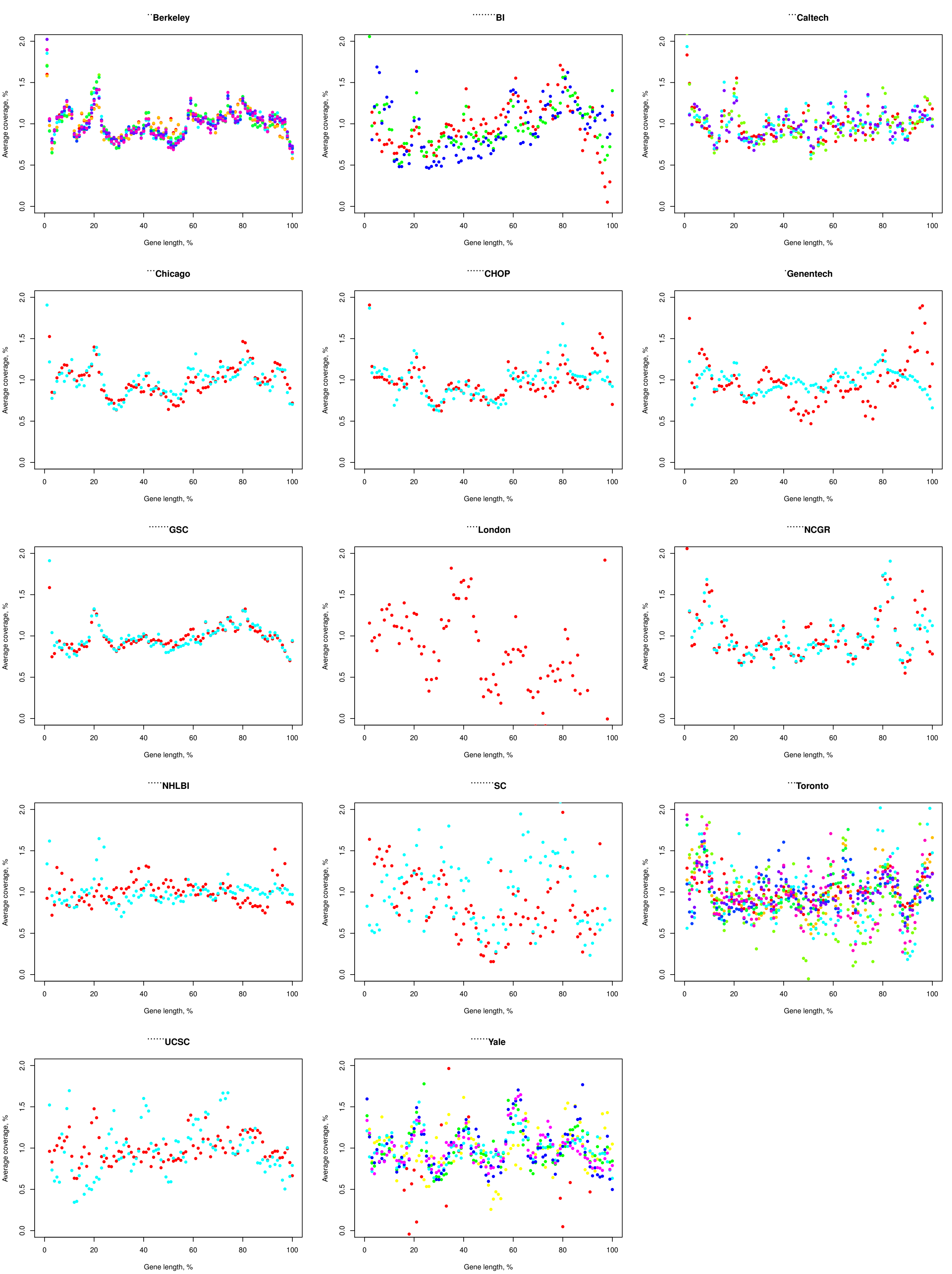

Supplement: Additional file 5 — Supplementary figure S5. Single-exon gene coverage distribution over gene length after normalization for mappability profiles. Points of different color represent different experiments grouped by laboratory. [file 1471-2105-13-S6-S4-S5.pdf]
