# Supplementary material for: Biases in read coverage demonstrated by interlaboratory and interplatform comparison of 117 mRNA and genome sequencing experiments
Source: BMC Bioinformatics. 2012 Apr 19;13(Suppl 6):S4. doi: 10.1186/1471-2105-13-S6-S4 (PMC3358657; doi:10.1186/1471-2105-13-S6-S4)
Supplement: Additional file 4 — Supplementary figure S4. Correlation of per-nucleotide coverage profiles between all pairs of sequencing experiments normalized for the 5' - 3' coverage non-uniformity. Notations are similar to those in Figure 1. [file 1471-2105-13-S6-S4-S4.pdf]

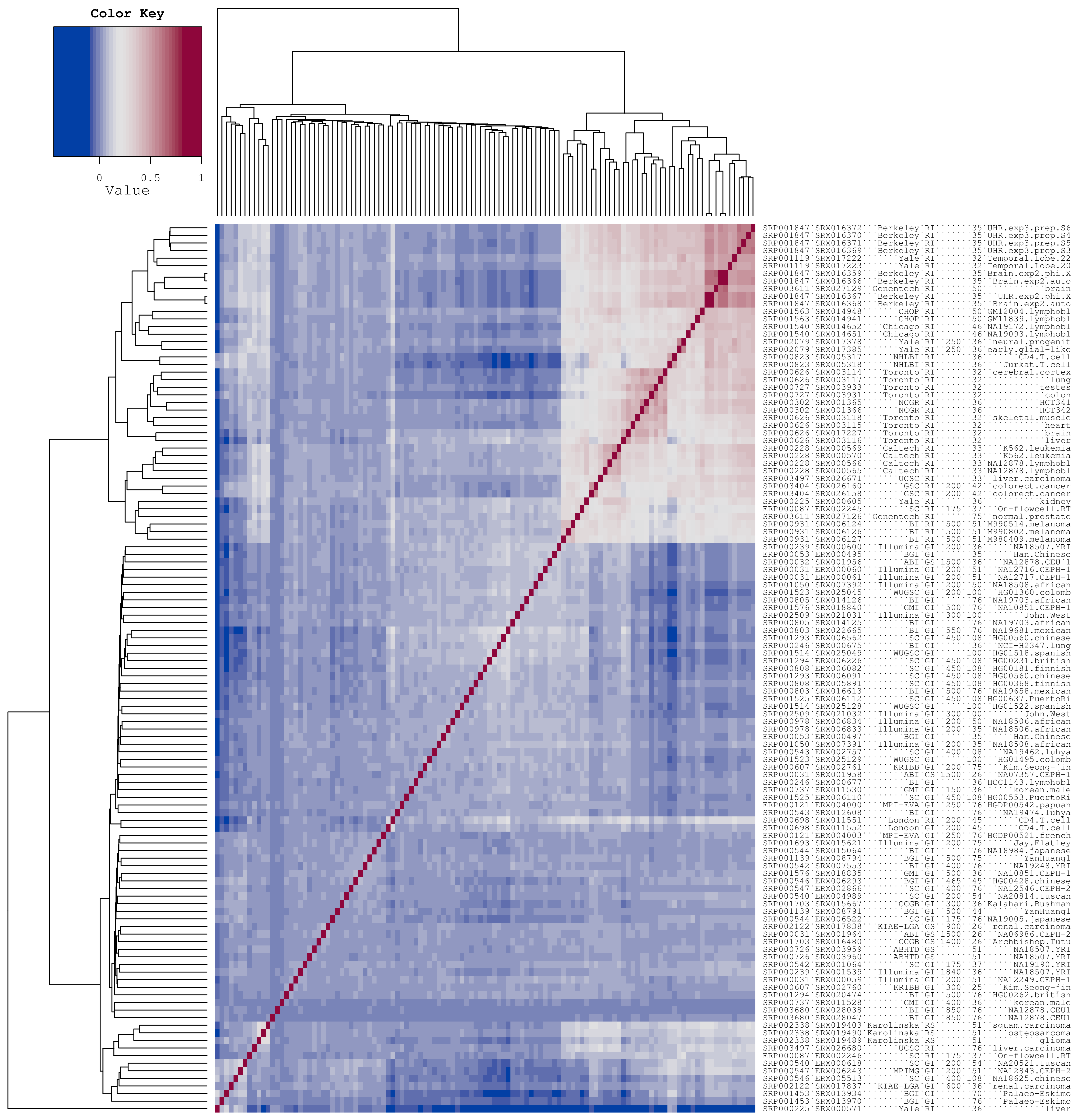

Color Key
0 0.5 1
Value
SRP001847 SRX016372 Berkeley RI 35 UHR.exp3.prep.S6
SRP001847 SRX016370 Berkeley RI 35 UHR.exp3.prep.S4
SRP001847 SRX016371 Berkeley RI 35 UHR.exp3.prep.S5
SRP001847 SRX016369 Berkeley RI 35 UHR.exp3.prep.S3
SRP001119 SRX017222 Yale RI 32 Temporal.Lobe.22
SRP001119 SRX017223 Yale RI 32 Temporal.Lobe.20
SRP001847 SRX016359 Berkeley RI 35 Brain.exp2.phi.X
SRP001847 SRX016366 Berkeley RI 35 Brain.exp2.auto
SRP003611 SRX027129 Genentech RI 50 brain
SRP001847 SRX016367 Berkeley RI 35 UHR.exp2.phi.X
SRP001847 SRX016368 Berkeley RI 35 Brain.exp2.auto
SRP001563 SRX014948 CHOP RI 50 GM12004.lymphobl
SRP001563 SRX014941 CHOP RI 50 GM11839.lymphobl
SRP001540 SRX014652 Chicago RI 46 NA19172.lymphobl
SRP001540 SRX014651 Chicago RI 46 NA19093.lymphobl
SRP002079 SRX017378 Yale RI 250 36 neural.progenit
SRP002079 SRX017385 Yale RI 250 36 early.glial-like
SRP000823 SRX005317 NHLBI RI 36 CD4.T.cell
SRP000823 SRX005318 NHLBI RI 36 Jurkat.T.cell
SRP000626 SRX003114 Toronto RI 32 cerebral.cortex
SRP000626 SRX003117 Toronto RI 32 lung
SRP000727 SRX003933 Toronto RI 32 testes
SRP000727 SRX003931 Toronto RI 32 colon
SRP000302 SRX001365 NCGR RI 36 HCT341
SRP000302 SRX001366 NCGR RI 36 HCT342
SRP000626 SRX003118 Toronto RI 32 skeletal.muscle
SRP000626 SRX003115 Toronto RI 32 heart
SRP000626 SRX017227 Toronto RI 32 brain
SRP000626 SRX003116 Toronto RI 32 liver
SRP000228 SRX000569 Caltech RI 33 K562.leukemia
SRP000228 SRX000570 Caltech RI 33 K562.leukemia
SRP000228 SRX000566 Caltech RI 33 NA12878.lymphobl
SRP000228 SRX000565 Caltech RI 33 NA12878.lymphobl
SRP003497 SRX026671 UCSC RI 33 liver.carcinoma
SRP003404 SRX026160 GSC RI 200 42 colorect.cancer
SRP003404 SRX026158 GSC RI 200 42 colorect.cancer
SRP000225 SRX000605 Yale RI 36 kidney
ERP000087 ERX002245 SC RI 175 37 On-flowcell.RT
SRP003611 SRX027126 Genentech RI 75 normal.prostate
SRP000931 SRX006124 BI RI 500 51 M990514.melanoma
SRP000931 SRX006126 BI RI 500 51 M990802.melanoma
SRP000931 SRX006127 BI RI 500 51 M980409.melanoma
SRP000239 SRX000600 Illumina GI 200 36 NA18507.YRI
ERP000053 ERX000495 BGI GI 35 Han.Chinese
SRP000032 SRX001956 ABI GS 1500 36 NA12878.CEU 1
SRP000031 ERX000060 Illumina GI 200 51 NA12716.CEPH-1
SRP000031 ERX000061 Illumina GI 200 51 NA12717.CEPH-1
SRP001050 SRX007392 Illumina GI 200 50 NA18508.african
SRP001523 SRX025045 WUGSC GI 200 100 HG01360.colomb
SRP000805 SRX014126 BI GI 76 NA19703.african
SRP001576 SRX018840 GMI GI 500 76 NA10851.CEPH-1
SRP002509 SRX021031 Illumina GI 300 100 John.West
SRP000805 SRX014125 BI GI 76 NA19703.african
SRP000803 SRX022665 BI GI 550 76 NA19681.mexican
SRP001293 ERX006562 SC GI 450 108 HG00560.chinese
SRP000246 SRX000675 BI GI 36 NCI-H2347.lung
SRP001514 SRX025049 WUGSC GI 100 HG01518.spanish
SRP001294 ERX006226 SC GI 450 108 HG00231.british
SRP000808 ERX006082 SC GI 450 108 HG00181.finnish
SRP001293 ERX006091 SC GI 450 108 HG00560.chinese
SRP000808 ERX005891 SC GI 450 108 HG00368.finnish
SRP000803 SRX016613 BI GI 500 76 NA19658.mexican
SRP001525 ERX006112 SC GI 450 108 HG00637.PuertoRi
SRP001514 SRX025128 WUGSC GI 100 HG01522.spanish
SRP002509 SRX021032 Illumina GI 300 100 John.West
SRP000978 SRX006834 Illumina GI 200 50 NA18506.african
SRP000978 SRX006833 Illumina GI 200 35 NA18506.african
ERP000053 ERX000497 BGI GI 35 Han.Chinese
SRP001050 SRX007391 Illumina GI 200 35 NA18508.african
SRP000543 ERX002757 SC GI 400 108 NA19462.luhya
SRP001523 SRX025129 WUGSC GI 100 HG01495.colomb
SRP000607 SRX002761 KRIBB GI 200 75 Kim.Seong-jin
SRP000031 SRX001958 ABI GS 1500 26 NA07357.CEPH-1
SRP000246 SRX000677 BI GI 36 HCC1143.lymphobl
SRP000737 SRX011530 GMI GI 150 36 korean.male
SRP001525 ERX006110 SC GI 450 108 HG00553.PuertoRi
ERP000121 ERX004000 MPI-EVA GI 250 76 HGDP00542.papuan
SRP000543 SRX012608 BI GI 76 NA19474.luhya
SRP000698 SRX011551 London RI 200 45 CD4.T.cell
SRP000698 SRX011552 London GI 200 45 CD4.T.cell
ERP000121 ERX004003 MPI-EVA GI 250 76 HGDP00521.french
SRP001693 SRX015621 Illumina GI 200 75 Jay.Flatley
SRP000544 SRX015064 BI GI 76 NA18984.japanese
SRP001139 SRX008794 BGI GI 500 75 YanHuang1
SRP000542 SRX007553 BI GI 400 76 NA19248.YRI
SRP001576 SRX018835 GMI GI 500 36 NA10851.CEPH-1
SRP000546 ERX006293 BGI GI 465 45 HG00428.chinese
SRP000547 ERX002866 SC GI 400 76 NA12546.CEPH-2
SRP000540 ERX004989 SC GI 200 54 NA20814.tuscan
SRP001703 SRX015667 CCGB GI 300 36 Kalahari.Bushman
SRP001139 SRX008791 BGI GI 500 44 YanHuang1
SRP000544 ERX006522 SC GI 175 76 NA19005.japanese
SRP002122 SRX017838 KIAE-LGA GS 900 26 renal.carcinoma
SRP000031 SRX001964 ABI GS 1500 26 NA06986.CEPH-2
SRP001703 SRX016480 CCGB GS 1400 26 Archbishop.Tutu
SRP000726 SRX003959 ABHTD GS 51 NA18507.YRI
SRP000726 SRX003960 ABHTD GS 51 NA18507.YRI
SRP000542 ERX001064 SC GI 175 37 NA19190.YRI
SRP000239 SRX001539 Illumina GI 1840 36 NA18507.YRI
SRP000031 ERX000059 Illumina GI 200 51 NA12249.CEPH-1
SRP000607 SRX002760 KRIBB GI 300 25 Kim.Seong-jin
SRP001294 SRX020474 BI GI 500 76 HG00262.british
SRP000737 SRX011528 GMI GI 400 36 korean.male
SRP003680 SRX028038 BI GI 850 76 NA12878.CEU1
SRP003680 SRX028047 BI GI 850 76 NA12878.CEU1
SRP002338 SRX019403 Karolinska RS 51 squam.carcinoma
SRP002338 SRX019490 Karolinska RS 51 osteosarcoma
SRP002338 SRX019489 Karolinska RS 51 glioma
SRP003497 SRX026680 UCSC RI 76 liver.carcinoma
ERP000087 ERX002246 SC RI 175 37 On-flowcell.RT
SRP000540 ERX000618 SC GI 200 54 NA20521.tuscan
SRP000547 ERX006243 MPIMG GI 200 51 NA12843.CEPH-2
SRP000546 ERX005513 SC GI 400 108 NA18625.chinese
SRP002122 SRX017837 KIAE-LGA GI 600 36 renal.carcinoma
SRP001453 SRX013934 BGI GI 70 Palaeo-Eskimo
SRP001453 SRX013970 BGI GI 76 Palaeo-Eskimo
SRP000225 SRX000571 Yale RI 36 liver
