# Supplementary material for: Biases in read coverage demonstrated by interlaboratory and interplatform comparison of 117 mRNA and genome sequencing experiments
Source: BMC Bioinformatics. 2012 Apr 19;13(Suppl 6):S4. doi: 10.1186/1471-2105-13-S6-S4 (PMC3358657; doi:10.1186/1471-2105-13-S6-S4)
Supplement: Additional file 6 — Supplementary figure S6. Additional information about 117 compared mRNA and genome high-throughput sequencing experiments. Experiments are clustered by similarity of single-exon gene coverage profiles as in Figure 1. [file 1471-2105-13-S6-S4-S6.pdf]

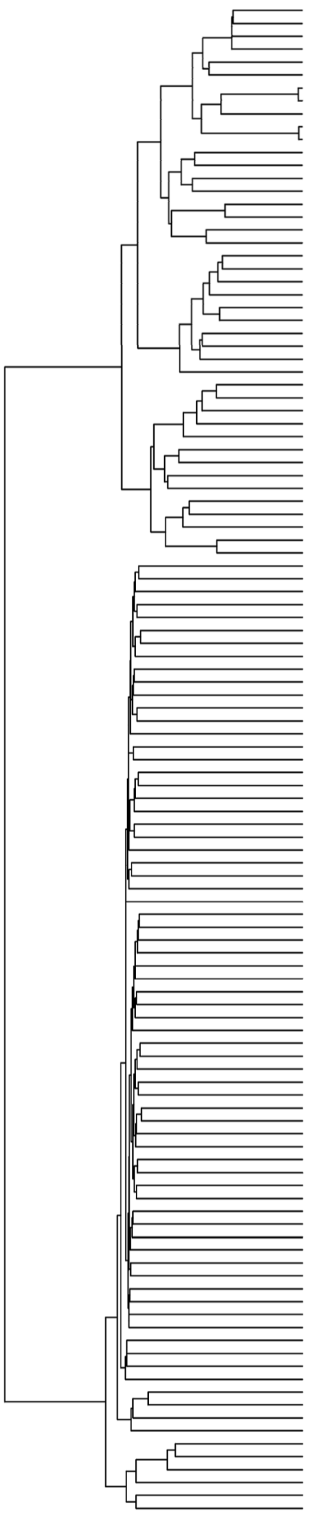

Study	Experiment	Institution	Institution full name	Project full name	Sample full name	Source	Insert	Read	Dataset size
SRP001847	SRX016371	Berkeley	University of California, Berkeley, USA	Evaluation of Statistical Methods for Normalization and Differential Expression in mRNA-Seq Experiments	MAQC UHR exp 3 library prep S5	RI		35	1.6G*48%=0.8G
SRP001847	SRX016369	Berkeley	University of California, Berkeley, USA	Evaluation of Statistical Methods for Normalization and Differential Expression in mRNA-Seq Experiments	MAQC UHR exp 3 library prep S3	RI		35	1.6G*49%=0.8G
SRP001847	SRX016370	Berkeley	University of California, Berkeley, USA	Evaluation of Statistical Methods for Normalization and Differential Expression in mRNA-Seq Experiments	MAQC UHR exp 3 library prep S4	RI		35	1.4G*49%=0.7G
SRP001847	SRX016372	Berkeley	University of California, Berkeley, USA	Evaluation of Statistical Methods for Normalization and Differential Expression in mRNA-Seq Experiments	MAQC UHR exp 3 library prep S6	RI		35	1.6G*47%=0.7G
SRP001119	SRX017222	Yale	Yale University, New Haven, USA	Developmental changes in human neocortical transcriptome revealed by RNA-seq	Female Anterior Temporal Lobe from brain tissue, 22 weeks gestation	RI		32	1.7G*53%=0.9G
SRP001119	SRX017223	Yale	Yale University, New Haven, USA	Developmental changes in human neocortical transcriptome revealed by RNA-seq	Female Anterior Temporal Lobe from brain tissue, 20 weeks gestation	RI		32	0.9G*45%=0.4G
SRP001847	SRX016359	Berkeley	University of California, Berkeley, USA	Evaluation of Statistical Methods for Normalization and Differential Expression in mRNA-Seq Experiments	MAQC Brain exp 2 using phi X control lane	RI		35	1.6G*51%=0.8G
SRP001847	SRX016366	Berkeley	University of California, Berkeley, USA	Evaluation of Statistical Methods for Normalization and Differential Expression in mRNA-Seq Experiments	MAQC Brain exp 2 using auto calibration	RI		35	1.6G*53%=0.8G
SRP003611	SRX027129	Genentech	Genentech, Inc., San Francisco, USA	Deep transcriptional sequencing analysis of human prostate adenocarcinoma and reference samples	Brain reference	RI		50	1.2G*58%=0.7G
SRP001847	SRX016368	Berkeley	University of California, Berkeley, USA	Evaluation of Statistical Methods for Normalization and Differential Expression in mRNA-Seq Experiments	MAQC Brain exp 2 using auto calibration	RI		35	1.8G*47%=0.8G
SRP001847	SRX016367	Berkeley	University of California, Berkeley, USA	Evaluation of Statistical Methods for Normalization and Differential Expression in mRNA-Seq Experiments	MAQC UHR exp 2 using phi X control lane	RI		35	1.8G*46%=0.8G
SRP001563	SRX014948	CHOP	Children's Hospital of Philadelphia, USA	RNA-seq of human immmortalized B-cells	Blood B-Lymphocite GM12004 cell line, female	RI		50	1.4G*43%=0.6G
SRP001563	SRX014941	CHOP	Children's Hospital of Philadelphia, USA	RNA-seq of human immmortalized B-cells	Blood B-Lymphocite GM11839 cell line, male	RI		50	1.4G*42%=0.5G
SRP001540	SRX014652	Chicago	Department of human genetics, University of Chicago, USA	Understaning mechanisms underlying human gene expression variation with RNA sequencing	HapMap Yoruba individual NA19172, lymphoblastoid cell line	RI		46	0.5G*54%=0.2G
SRP001540	SRX014651	Chicago	Department of human genetics, University of Chicago, USA	Understaning mechanisms underlying human gene expression variation with RNA sequencing	HapMap Yoruba individual NA19093, lymphoblastoid cell line	RI		46	0.5G*56%=0.3G
SRP002079	SRX017378	Yale	Yale University, New Haven, USA	Dynamic transcriptomes during neural differentiation of human embryonic stem cells	developmental stage: neural progenitors	RI	250	36	1.2G*48%=0.6G
SRP002079	SRX017385	Yale	Yale University, New Haven, USA	Dynamic transcriptomes during neural differentiation of human embryonic stem cells	developmental stage: early glial-like	RI	250	36	1.5G*42%=0.6G
SRP000823	SRX005317	NHLBI	National Heart, Lung and Blood Institute, NIH, Bethesda, USA	Detection of single nucleotide variations in expressed exons of the human genome using RNA-Seq	CD4+ T cell polyA purified mRNA	RI		36	1.4G*34%=0.5G
SRP000823	SRX005318	NHLBI	National Heart, Lung and Blood Institute, NIH, Bethesda, USA	Detection of single nucleotide variations in expressed exons of the human genome using RNA-Seq	Jurkat T cell polyA purified mRNA	RI		36	1.5G*37%=0.5G
SRP000302	SRX001365	NCGR	National Center for Genome Resources, Santa Fe, USA	Alternative Isoform Regulation in Human Tissue Transcriptomes	HCT341 library	RI		36	0.9G*49%=0.4G
SRP000302	SRX001366	NCGR	National Center for Genome Resources, Santa Fe, USA	Alternative Isoform Regulation in Human Tissue Transcriptomes	HCT342 library	RI		36	0.8G*51%=0.4G
SRP000626	SRX003114	Toronto	University of Toronto, Canada	Deep surveying of alternative splicing complexity	Cerebral cortex tissue	RI		32	0.5G*52%=0.5G
SRP000626	SRX003117	Toronto	University of Toronto, Canada	Deep surveying of alternative splicing complexity	Lung tissue	RI		32	0.8G*45%=0.3G
SRP000727	SRX003933	Toronto	University of Toronto, Canada	Alternative Isoform Regulation in Human Tissue Transcriptomes	testes tissue	RI		32	0.9G*57%=0.5G
SRP000727	SRX003931	Toronto	University of Toronto, Canada	Alternative Isoform Regulation in Human Tissue Transcriptomes	colon tissue	RI		32	0.9G*49%=0.4G
SRP000626	SRX003118	Toronto	University of Toronto, Canada	Deep surveying of alternative splicing complexity	Skeletal muscle tissue	RI		32	0.7G*50%=0.3G
SRP000626	SRX003115	Toronto	University of Toronto, Canada	Deep surveying of alternative splicing complexity	Heart tissue	RI		32	0.6G*38%=0.2G
SRP000626	SRX017227	Toronto	University of Toronto, Canada	Deep surveying of alternative splicing complexity	Brain tissue	RI		32	0.5G*50%=0.2G
SRP000626	SRX003116	Toronto	University of Toronto, Canada	Deep surveying of alternative splicing complexity	Liver tissue	RI		32	0.5G*51%=0.3G
SRP000228	SRX000569	Caltech	California Institute of Technology, Pasadena, USA	Homo sapiens transcriptome analysis by RNA-Seq for ENCODE project	K562 chronic myelogenous leukemia cell line, female	RI		33	1.5G*29%=0.4G
SRP000228	SRX000570	Caltech	California Institute of Technology, Pasadena, USA	Homo sapiens transcriptome analysis by RNA-Seq for ENCODE project	K562 chronic myelogenous leukemia cell line, female	RI		33	1.6G*24%=0.4G
SRP000228	SRX000566	Caltech	California Institute of Technology, Pasadena, USA	Homo sapiens transcriptome analysis by RNA-Seq for ENCODE project	HapMap individual NA12878, lymphoblastoid cell line, female	RI		33	1.5G*23%=0.3G
SRP000228	SRX000565	Caltech	California Institute of Technology, Pasadena, USA	Homo sapiens transcriptome analysis by RNA-Seq for ENCODE project	HapMap individual NA12878, lymphoblastoid cell line, female	RI		33	1.6G*20%=0.3G
SRP003497	SRX026671	UCSC	UCSC ENCODE, Santa Cruz, USA	ENCODE Caltech RNA-seq	Liver carcinoma cell line HepG2, male	RI		33	1.4G*21%=0.3G
SRP000225	SRX000605	Yale	Yale University, New Haven, USA	RNASeq: the death Knell of expression arrays	Kidney, male	RI		36	1.4G*25%=0.3G
SRP000225	SRX000571	Yale	Yale University, New Haven, USA	RNASeq: the death Knell of expression arrays	Liver, male	RI		36	0.5G*25%=0.5G
SRP003611	SRX027126	Genentech	Genentech, Inc., San Francisco, USA	Deep transcriptional sequencing analysis of human prostate adenocarcinoma and reference samples	Adjacent normal prostate	RI		75	1.3G*56%=0.7G
ERP000087	ERX002245	SC	The Wellcome Trust Sanger Institute, Cambridge, UK	On-flowcell reverse transcription facilitates amplification-free, strand-specific, transcriptome sequencing	Flowcell-T-RnD-sc-Flowcell-T-RnD-sc-HP-S1-mRNA-250pM-1	RI	175	37	1.4G*32%=0.4G
SRP000931	SRX006124	BI	Broad Institute, MIT, Cambridge, USA	Melanoma Transcriptome	Melanoma, individual M990514	RI	500	51	1.6G*36%=0.5G
SRP000931	SRX006126	BI	Broad Institute, MIT, Cambridge, USA	Melanoma Transcriptome	Melanoma, individual M990802	RI	500	51	1.6G*32%=0.5G
SRP000931	SRX006127	BI	Broad Institute, MIT, Cambridge, USA	Melanoma Transcriptome	Melanoma, individual M980409	RI	500	51	1.6G*29%=0.4G
SRP003404	SRX026160	GSC	Genome Sciences Centre, British Columbia Cancer Agency, Vancouver, Canada	Comparison of 5-FU sensitive and resistant colorectal cancer cell lines	Colorectal 5-FU resistant cancer cell line MIP/5FU, liver metastasis	RI	200	42	1.5G*49%=0.7G
SRP003404	SRX026158	GSC	Genome Sciences Centre, British Columbia Cancer Agency, Vancouver, Canada	Comparison of 5-FU sensitive and resistant colorectal cancer cell lines	Colorectal 5-FU sensitive cancer cell line MIP101, liver metastasis	RI	200	42	1.5G*50%=0.7G
SRP001139	SRX008794	BGI	Beijing Genomics Institute, Shenzhen, China	YanHuang1 denovo assemble project	YanHuang1 chinese individual, male	GI	500	75	1.4G*75%=1.0G
SRP000542	SRX007553	BI	Broad Institute, MIT, Cambridge, USA	Full production for the 1000 genomes (YRI) Yoruba sequencing	HapMap individual NA19248, YRI, male	GI	400	76	1.4G*70%=0.9G
SRP000544	SRX015064	BI	Broad Institute, MIT, Cambridge, USA	Full production for the 1000 genomes (JPT) Japanese in Tokyo	HapMap individual NA18984, Japanese	GI		76	1.2G*64%=0.7G
ERP000121	ERX004003	MPI-EVA	Max Planck Institute for Evolutionary Anthropology, Leipzig, Germany	Human Genome Diversity Project	MMS8 HGDP library of a French individual	GI	250	76	1.4G*68%=0.9G
SRP001693	SRX015621	Illumina	Illumina	PG0000001 Individual human genome sequencing project	Peripheral blood lymphocytes of male individual Jay Flatley	GI	200	75	1.5G*72%=1.0G
SRP001576	SRX018835	GMI	Genomic Medicine Institute, Seoul National University, Korea	Whole genome sequencing of human HapMap individual NA10851	HapMap individual NA10851, CEPH-1, male	GI	500	36	1.5G*63%=0.9G
SRP000546	ERX006293	BGI	Beijing Genomics Institute, Shenzhen, China	Full production for the 1000 genomes Han Chinese in Beijing	HapMap individual HG00428, Southern Han Chinese, female	GI	465	45	1.5G*68%=1.0G
SRP000547	SRX002866	SC	The Wellcome Trust Sanger Institute, Cambridge, UK	Full production for the 1000 genomes CEPH sequencing	HapMap individual NA12546, CEPH - 2, male	GI	400	76	1.4G*71%=0.9G
SRP000540	ERX004989	SC	The Wellcome Trust Sanger Institute, Cambridge, UK	Full production for the 1000 genomes Toscan sequencing	HapMap individual NA20814, Tuscan, male	GI	200	54	1.4G*65%=0.9G
SRP002122	SRX017837	KIAE-LGA	Russian Research Centre Kurchatov Institute, Moscow, Russia	Whole genome sequencing of human (russian male)	Russian male individual with renal cell carcinoma	GI	600	36	2.7G*46%=1.2G
SRP001703	SRX015667	CCGB	Center for Comparative Genomics and Bioinformatics, Pennsylvania State University, USA	The genetic structure of the indigenous hunter-gatherer peoples of Southern Africa	Blood of !Gubi Kalahari Bushman, male	GI	300	36	0.8G*67%=0.5G
SRP000542	ERX001064	SC	The Wellcome Trust Sanger Institute, Cambridge, UK	Full production for the 1000 genomes (YRI) Yoruba sequencing	HapMap individual NA19190, YRI, female	GI	175	37	1.3G*68%=0.9G
SRP000239	SRX001539	Illumina	Illumina	Human genome sequencing of an African male individual	HapMap individual NA18507, male	GI	1840	36	1.4G*60%=0.8G
SRP000544	ERX006522	SC	The Wellcome Trust Sanger Institute, Cambridge, UK	Full production for the 1000 genomes (JPT) Japanese in Tokyo	HapMap individual NA19005, Japanese - 1, male	GI	175	76	1.7G*68%=1.1G
SRP001139	SRX008791	BGI	Beijing Genomics Institute, Shenzhen, China	YanHuang1 denovo assemble project	YanHuang1 chinese individual, male	GI	500	44	1.5G*73%=1.0G
SRP000737	SRX011530	GMI	Genomic Medicine Institute, Seoul National University, Korea	First Altaic Korean Individual Sequencing	The Altaic Korean Individual	GI	150	36	1.4G*37%=0.5G
SRP000031	SRX001964	ABI	ABI Lifetech	1000Genomes Project Pilot 1	HapMap individual NA06986, CEPH-2, male	GS	1500	26	1.5G*42%=0.6G
SRP002122	SRX017838	KIAE-LGA	Russian Research Centre Kurchatov Institute, Moscow, Russia	Whole genome sequencing of human (russian male)	Russian male individual with renal cell carcinoma	GS	900	26	3.2G*34%=1.0G
SRP000031	SRX001958	ABI	ABI Lifetech	1000Genomes Project Pilot 1	HapMap individual NA07357, CEPH-1, male	GS	1500	26	1.5G*39%=0.6G
SRP001703	SRX016480	CCGB	Center for Comparative Genomics and Bioinformatics, Pennsylvania State University, USA	The genetic structure of the indigenous hunter-gatherer peoples of Southern Africa	Blood of Archbishop Tutu Bushman, male	GS	1400	26	0.8G*27%=0.8G
SRP000726	SRX003959	ABHTD	Applied Biosystems High Throughput Discovery	Genome variability analysis of Yoruba in Ibadan, Nigeria	HapMap individual NA18507, YRI, male	GS		51	1.6G*37%=0.5G
SRP000726	SRX003960	ABHTD	Applied Biosystems High Throughput Discovery	Genome variability analysis of Yoruba in Ibadan, Nigeria	HapMap individual NA18507, YRI, male	GS		51	1.4G*13%=0.1G
SRP000246	SRX000677	BI	Broad Institute, MIT, Cambridge, USA	High-resolution mapping of copy-number alterations	Lymphoblastoid cell line HCC1143	GI		36	1.2G*22%=0.2G
SRP000607	SRX002760	KRIBB	Korea Reseach Institute of Bioscience and BioTechnology, Daejeon, South Korea	Korean individual genome sequence	Korean cancer research specialist Kim Seong-jin	GI	300	25	1.4G*66%=0.9G
SRP001294	SRX020474	BI	Broad Institute, MIT, Cambridge, USA	Full production for the 1000 genomes of British individuals from England and Scotland	1000 genomes individual HG00262, british, female	GI	500	76	1.7G*69%=1.1G
SRP000803	SRX016613	BI	Broad Institute, MIT, Cambridge, USA	1000 Genomes Low Coverage Sequencing - Mexican-American Population	HapMap individual NA19658, mexican, male	GI	500	76	1.6G*51%=0.8G
SRP000698	SRX011552	London	Barts and The London	Genome-wide analysis of allelic expression imbalance	CD4 T cell Individual 5 Activated	GI	200	45	1.4G*87%=1.2G
SRP000978	SRX006834	Illumina	Illumina	Human genome sequencing of an African male individual	HapMap individual NA18506, african, male	GI	200	50	1.6G*71%=1.1G
SRP000031	ERX000060	Illumina	Illumina	1000Genomes Project Pilot 1	HapMap individual NA12716, CEPH-1, male	GI	200	51	1.5G*73%=1.1G
SRP000239	SRX000600	Illumina	Illumina	Human genome sequencing of an African male individual	HapMap individual NA18507, male	GI	200	36	1.3G*68%=0.8G
SRP000978	SRX006833	Illumina	Illumina	Human genome sequencing of an African male individual	HapMap individual NA18506, african, male	GI	200	35	1.2G*69%=0.9G
SRP001050	SRX007391	Illumina	Illumina	Human genome sequencing of an African female individual	HapMap individual NA18508, african, female	GI	200	35	1.3G*69%=0.9G
ERP000053	ERX000497	BGI	Beijing Genomics Institute, Shenzhen, China	The diploid genome sequence of an Asian individual	Healthy Han Chinese individual	GI		35	1.4G*59%=0.8G
SRP000032	SRX001956	ABI	ABI Lifetech	1000Genomes Project Pilot 2	HapMap individual NA12878, CEU1, female	GS	1500	36	1.5G*27%=0.4G
SRP000031	ERX000061	Illumina	Illumina	1000Genomes Project Pilot 1	HapMap individual NA12717, CEPH-1, female	GI	200	51	1.4G*74%=1.0G
ERP000053	ERX000495	BGI	Beijing Genomics Institute, Shenzhen, China	The diploid genome sequence of an Asian individual	Healthy Han Chinese individual	GI		35	1.4G*70%=1.0G
SRP000031	ERX000059	Illumina	Illumina	1000Genomes Project Pilot 1	HapMap individual NA12249, CEPH-1, female	GI	200	51	1.3G*73%=0.9G
SRP001576	SRX018840	GMI	Genomic Medicine Institute, Seoul National University, Korea	Whole genome sequencing of human HapMap individual NA10851	HapMap individual NA10851, CEPH-1, male	GI	500	76	3.4G*65%=2.2G
SRP001523	SRX025045	WUGSC	Washington University Genome Science Center, USA	Full production for the 1000 genomes Colombian in Medellin, Colombia	1000 genomes individual HG01360, colombian, female	GI	200	100	1.6G*83%=1.6G
SRP000805	SRX014126	BI	Broad Institute, MIT, Cambridge, USA	1000 Genomes Low Coverage Sequencing - African-American Population	HapMap individual NA19703, african, male	GI		76	1.4G*71%=0.9G
SRP001514	SRX025128	WUGSC	Washington University Genome Science Center, USA	Full production for the 1000 genomes Iberian populations in Spain	1000 genomes individual HG01522, spanish, female	GI		100	2.7G*80%=2.1G
SRP002509	SRX021032	Illumina	Illumina	PG0000021 Individual human genome sequencing project	Peripheral blood lymphocytes of male individual John West	GI	300	100	1.5G*76%=1.1G
SRP001514	SRX025049	WUGSC	Washington University Genome Science Center, USA	Full production for the 1000 genomes Iberian populations in Spain	1000 genomes individual HG01518, spanish, male	GI		100	1.6G*81%=1.6G
SRP001293	ERX006562	SC	The Wellcome Trust Sanger Institute, Cambridge, UK	Full production for the 1000 genomes African ancestry from Southern Han Chinese population	1000 genomes individual HG00560, chinese, female	GI	450	108	1.5G*72%=1.0G
SRP001050	SRX007392	Illumina	Illumina	Human genome sequencing of an African female individual	HapMap individual NA18508, african, female	GI	200	50	1.5G*72%=1.1G
SRP001294	ERX006226	SC	The Wellcome Trust Sanger Institute, Cambridge, UK	Full production for the 1000 genomes of British individuals from England and Scotland	1000 genomes individual HG00231, british, female	GI	450	108	1.5G*78%=1.1G
SRP000805	SRX014125	BI	Broad Institute, MIT, Cambridge, USA	1000 Genomes Low Coverage Sequencing - African-American Population	HapMap individual NA19703, african, male	GI		76	1.5G*71%=1.0G
SRP002509	SRX021031	Illumina	Illumina	PG0000021 Individual human genome sequencing project	Peripheral blood lymphocytes of male individual John West	GI	300	100	1.7G*73%=1.2G
SRP000607	SRX002761	KRIBB	Korea Reseach Institute of Bioscience and BioTechnology, Daejeon, South Korea	Korean individual genome sequence	Korean cancer research specialist Kim Seong-jin	GI	200	75	1.5G*77%=1.1G
SRP001523	SRX025129	WUGSC	Washington University Genome Science Center, USA	Full production for the 1000 genomes Colombian in Medellin, Colombia	1000 genomes individual HG01495, colombian, female	GI		100	2.5G*82%=2.0G
ERP000121	ERX004000	MPI-EVA	Max Planck Institute for Evolutionary Anthropology, Leipzig, Germany	Human Genome Diversity Project	MMS5 HGDP library of a Papuan (PNG) individual	GI	250	76	1.8G*61%=1.0G
SRP000543	ERX002757	SC	The Wellcome Trust Sanger Institute, Cambridge, UK	Full production for the 1000 genomes Luhya sequencing	HapMap individual NA19462, Luhya, female	GI	400	108	1.4G*69%=0.9G
SRP001525	ERX006112	SC	The Wellcome Trust Sanger Institute, Cambridge, UK	Full production for the 1000 genomes Puerto Rican in Puerto Rico	1000 genomes individual HG00637, puertorican, male	GI	450	108	1.6G*78%=1.2G
SRP000543	SRX012608	BI	Broad Institute, MIT, Cambridge, USA	Full production for the 1000 genomes Luhya sequencing	HapMap individual NA19474, Luhya, female	GI		76	1.8G*21%=0.3G
SRP000808	ERX005891	SC	The Wellcome Trust Sanger Institute, Cambridge, UK	Full production for the 1000 genomes Finnish HapMap population sequencing	1000 genomes individual HG00368, finnish, female	GI	450	108	1.4G*73%=1.0G
SRP001525	ERX006110	SC	The Wellcome Trust Sanger Institute, Cambridge, UK	Full production for the 1000 genomes Puerto Rican in Puerto Rico	1000 genomes individual HG00553, puertorican, male	GI	450	108	1.5G*78%=1.1G
SRP000246	SRX000675	BI	Broad Institute, MIT, Cambridge, USA	High-resolution mapping of copy-number alterations	Lung cell line NCI-H2347	GI		36	1.0G*31%=0.3G
SRP001293	ERX006091	SC	The Wellcome Trust Sanger Institute, Cambridge, UK	Full production for the 1000 genomes African ancestry from Southern Han Chinese population	1000 genomes individual HG00560, chinese, female	GI	450	108	1.5G*79%=1.1G
SRP000808	ERX006082	SC	The Wellcome Trust Sanger Institute, Cambridge, UK	Full production for the 1000 genomes Finnish HapMap population sequencing	1000 genomes individual HG00181, finnish, male	GI	450	108	1.4G*65%=0.9G
SRP000803	SRX022665	BI	Broad Institute, MIT, Cambridge, USA	1000 Genomes Low Coverage Sequencing - Mexican-American Population	HapMap individual NA19681, mexican, female	GI	550	76	1.3G* 9%=0.1G
SRP003680	SRX028038	BI	Broad Institute, MIT, Cambridge, USA	Human NA12878 genome sequenced using multiple library types	HapMap individual NA12878, CEU-1, female	GI	850	76	1.7G* 0%=0.0G
SRP003680	SRX028047	BI	Broad Institute, MIT, Cambridge, USA	Human NA12878 genome sequenced using multiple library types	HapMap individual NA12878, CEU-1, female	GI	850	76	1.7G* 0%=0.0G
SRP000737	SRX011528	GMI	Genomic Medicine Institute, Seoul National University, Korea	First Altaic Korean Individual Sequencing	The Altaic Korean Individual	GI	400	36	1.2G*26%=0.3G
SRP001453	SRX013970	BGI	Beijing Genomics Institute, Shenzhen, China	Ancient human genome sequence of an extinct Palaeo-Eskimo	4000 year old male individual, hair	GI		76	1.5G* 6%=0.0G
SRP000540	ERX000618	SC	The Wellcome Trust Sanger Institute, Cambridge, UK	Full production for the 1000 genomes Toscan sequencing	HapMap individual NA20521, Tuscan, male	GI	200	54	1.4G*68%=0.9G
SRP000547	ERX006243	MPIMG	Max Planck Institute for Molecular Genetics, Berlin, Germany	Full production for the 1000 genomes CEPH sequencing	HapMap individual NA12843, CEPH - 2, female	GI	200	51	1.5G*65%=0.9G
SRP001453	SRX013934	BGI	Beijing Genomics Institute, Shenzhen, China	Ancient human genome sequence of an extinct Palaeo-Eskimo	4000 year old male individual, hair	GI		70	1.4G*19%=0.2G
SRP000546	ERX005513	SC	The Wellcome Trust Sanger Institute, Cambridge, UK	Full production for the 1000 genomes Han Chinese in Beijing	HapMap individual NA18625, Han Chinese - 2, female	GI	400	108	1.2G*67%=0.8G
SRP002338	SRX019403	Karolinska	Karolinska Institute, Stockholm, Sweden	Deep sequencing of the transcriptome of three human cell lines used in the Human Protein Atlas program	Squamous carcinoma cell line A431	RS		51	0.7G*17%=0.1G
SRP002338	SRX019490	Karolinska	Karolinska Institute, Stockholm, Sweden	Deep sequencing of the transcriptome of three human cell lines used in the Human Protein Atlas program	Osteosarcoma cell line U2-OS	RS		51	0.5G*16%=0.0G
SRP002338	SRX019489	Karolinska	Karolinska Institute, Stockholm, Sweden	Deep sequencing of the transcriptome of three human cell lines used in the Human Protein Atlas program	Glioma cell line U251	RS		51	0.5G*15%=0.0G
SRP003497	SRX026680	UCSC	UCSC ENCODE, Santa Cruz, USA	ENCODE Caltech RNA-seq	Liver carcinoma cell line HepG2, male	RI		76	1.4G*33%=0.4G
SRP000698	SRX011551	London	Barts and The London	Genome-wide analysis of allelic expression imbalance	CD4 T cell Individual 5 Activated	RI	200	45	1.5G*78%=1.1G
ERP000087	ERX002246	SC	The Wellcome Trust Sanger Institute, Cambridge, UK	On-flowcell reverse transcription facilitates amplification-free, strand-specific, transcriptome sequencing	Flowcell-T-RnD-sc-Flowcell-T-RnD-sc-HP-S1-mRNA-250pM-1	RI	175	37	1.3G*31%=0.4G
